# Flexible stabilization of the distal tibiofibular syndesmosis: clinical and biomechanical considerations: a review of the literature

**DOI:** 10.1007/s11751-012-0147-2

**Published:** 2012-10-25

**Authors:** Annick den Daas, Wouter J. van Zuuren, Stéphane Pelet, Arthur van Noort, Michel P. J. van den Bekerom

**Affiliations:** 1 Department of Orthopaedic Surgery, Spaarne Hospital, Spaarnepoort 1, PO Box 770, 2130 AT Hoofddorp, The Netherlands; 2 Department of Orthopaedic Surgery, Clinique Université Laval CHA-Pavillon Enfant-Jésus, 1401, 18 ème Rue, QC, G1J 1Z4 Canada

**Keywords:** Syndesmosis, Screw, Ankle fracture, Flexible implant, Instability, Biomechanical

## Abstract

Syndesmotic rupture is present in 10 % of ankle fractures and must be recognized and treated to prevent late complications. The method of fixation is classically rigid fixation with one or two screws. Knowledge of the biomechanics of the syndesmosis has led to the development of new dynamic implants to restore physiologic motion during walking. One of these implants is the suture-button system. The purpose of this paper is to review the orthopaedic trauma literature, both biomechanical and clinical, to present the current state of knowledge on the suture-button fixation and to put emphasis on the advantages and disadvantages of this technique. Two investigators searched the databases of Pubmed/Medline, Cochrane Clinical Trial Register and Embase independently. The search interval was from January 1980 to March 2011. The search keys comprised terms to identify articles on biomechanical and clinical issues of flexible fixation of syndesmotic ruptures. Ninety-nine publications met the search criteria. After filtering using the exclusion criteria, 11 articles (five biomechanical and six clinical) were available for review. The biomechanical studies involved 90 cadaveric ankles. The suture-button demonstrated good resistance to axial and rotational loads (equivalent to screws) and resistance to failure. Physiologic motion of the syndesmosis was restored in all directions. The clinical studies (149 ankles) demonstrated good functional results using the AOFAS score, indicating faster rehabilitation with flexible fixation than with screws. There were few complications. Preliminary results from the current literature support the use of suture-button fixation for syndesmotic ruptures. This method seems secure and safe. As there is no strong evidence for its use, prospective randomized controlled trials to compare the suture-button to the screw fixation for ankle syndesmotic ruptures are required.

## Introduction

Syndesmotic instability occurs in approximately 10 % of all ankle fractures and in 25 % of all ankle fractures that require surgery [20, 25]. The syndesmosis comprises the anterior and posterior inferior tibiofibular ligaments and a central interosseous ligament that extends more proximally as the interosseous membrane [2, 24]. Classic injuries associated with syndesmotic instability can be described as a pronation-external rotation (PER) type injury (according to Lauge-Hansen) or a Danis–Weber type C classification [14, 23]. Weber B fractures with medial injury, occurring after supination-external rotation injury (SER IV), can also be associated with syndesmotic instability. Pure ligamentous injuries to the syndesmosis also occur, typically with external rotation injuries in athletics. All these injuries fall under the same indication requiring syndesmotic fixation [21].

Poor functional outcomes and the development of osteoarthritis due to widening and chronic instability of the distal tibiofibular syndesmosis have led to the widespread practice of anatomical restoration and stabilization of the ankle mortise and syndesmosis [9, 11, 29, 41]. For the treatment of syndesmotic instability, the AO/ASIF-group recommends a tibiofibular transfixation screw [12]. Problems with this type of fixation have been reported and included the following: late syndesmotic widening after screw removal; screw loosening; screw breakage; the need for a second operation to remove the screw; and morbidity associated with prolonged immobilization [5, 9]. To avoid this need for removal, bioabsorbable syndesmotic screws have been proposed [40]. However, these may fail before healing is complete or may cause osteolysis [4] and, in case of infection, may be difficult or even impossible to remove.

The more recent development is the suture-button (flexible implant) with the potential advantage of preserving physiologic motion in the tibiofibular joint [18]. Rigid fixation with a screw eliminates this normal motion, potentially resulting in pain or hardware failure [32]. An ideal implant to stabilize the tibiofibular syndesmosis should allow early mobilization and be strong enough to maintain reduction in the syndesmosis [19]. These characteristics appear to be met in the suture-button but little is known about the short- and long-term results of this implant [40]. The objective of this review is to highlight the pros and cons of suture-button fixation for stabilizing the distal tibiofibular syndesmosis based on the current available literature (biomechanical and clinical). Answers to the following questions are sought: Is the suture-button secure? Is it easy to apply? Is it efficient? Is it cost-effective?

## Materials and methods

A literature search was conducted to identify studies in which patients were treated for syndesmotic instability with a suture-button. Inclusion criteria were age (over 18 years), acute syndesmotic instability (isolated and with associated fracture) and treatment with the suture-button technique. The suture-button fixation technique was defined as any stabilization technique for syndesmotic instability which is not static and allowed for some degree of tibiofibular movement. Examples of fixation hardware meeting this criterion include the TightRope (Arthrex, Naples, FL, US), ZipTight (Biomet Warsaw, Indiana, USA), and Endobutton (Smith & Nephew, Andover, MA, US). The exclusion criteria were as follows: animal studies, syndesmotic stabilization with other stabilization (static or fixed) methods and reports on fewer than two patients. Biomechanical and clinical studies were included (Tables 1, 2). Article language was restricted to English, German and Dutch. For this search, the following Boolean operators were used: ‘syndesmo*’ OR ‘tibiofibular’ AND ‘flexible fixation’ OR ‘suture’ OR ‘Acufex’ OR ‘Tightrope’ OR ‘Arthrex’ OR ‘Ziptight’ OR ‘EndoButton’. Literature was searched in the databases of Pubmed/Medline, Cochrane Clinical Trail Register and Embase from January 1980 to March 2011. Two investigators (AD and WZ) explored the databases independently. The articles with potentially applicable titles and/or abstracts were obtained and relevance was assessed. Potentially eligible articles were screened by two authors (AD and WZ) for applicability, and references of used publications were verified for additional studies meeting the inclusion criteria.

**Table 1:** Characteristics of mechanical studies

Study	*n*	Outcome	Method	Conclusion
Klitzman et al. [17]	8 fresh frozen human ankles	Syndesmotic gap Tibiofibular movement Laxity due to cycling	Cycling at submaximal loads in six-degrees-of-freedom-machine Dorsal/plantar flexion; internal/external rotation and inversion/eversion	Good alternative for syndesmotic fixation. More physiologic type of fixation and a good ability to maintain reduction in syndesmosis. No second surgery necessary
Soin et al. [35]	Ten pairs of cadaveric legs	Fibular translations and rotation	Axial compression, external rotation and combination Linear variable displacement transducer	Screws were closer to native ankle motion in AP and ML motions; Suture-button was closer to native fibular rotation
Forsythe et al. [10]	Ten fresh frozen cadaveric ankle pairs	Maintain syndesmotic reduction as compared to metallic screw	External rotation force on intact ankles and after dissecting the syndesmotic and deltoid ligaments	The fibre wire button was unable to maintain syndesmotic reduction in the ankle at any forces applied
Thornes et al. [37]	Sixteen embalmed cadaveric legs	Diastasis in suture-button versus 4 cortical screw	Generating an external rotation torque	Suture-endobutton fixation at least equals the performance of screw fixation
Miller et al. [22]	26 formalin-preserved cadaveric legs	Maximum load and displacement at failure in suture constructs and tricortical screws	Tested to failure along the axis of the repair apparatus. Screw versus suture at 2 and 5 cm above tibial plafond	Good alternative to internal fixation of ankle mortise instability due to syndesmotic rupture

**Table 2:** Characteristics of clinical studies

Study	*n*	Outcome	Method	Conclusion	Level of evidence
Cottom et al. [7]	50 25 Tightrope 25 screw	AOFAS, SF12	Single tightrope/double tightrope versus screw fixation	AOFAS and SF12 no significant difference between screw and tightrope, 6 months postoperatively	II
Thornes et al. [38]	32 16 screw 16 flexible fixation	AOFAS	4 cortices syndesmotic screw fixation versus suture-button fixation	AOFAS was significantly better in the suture-button group after 3 months and 1 year	III
Cottom et al. [6]	25	AOFAS, SF 12	Single tightrope/double tightrope	Method quick to perform. No complications, early weight-bearing, early return to daily living, sports and work	IV
Willmott et al. [42]	6	Radiological evaluation	5 single and one double tightrope	2/6 removed. One because tender swelling over button	IV
Thornes et al. [38]	12	AOFAS	Single tightrope fixation	No major complications, AOFAS mean 87 at FU at least 6 months, 8/8 returned to work in 3–16 week. Mean dorsiflexion 4.3 beyond neutral versus 8.7 contralateral	IV
de Groot et al. [8]	24	AOFAS	Single/double/triple tightrope	AOFAS 94 mean at last FU. (20 months) No major complications. 6 devices removed. 2× suture-button with subsidence Despite this no worse functional outcome	IV

Data extracted from clinical studies included operative technique, functional outcome measures, complications and follow-up. From the biomechanical studies, the methods of testing and biomechanical outcome were assessed. Recommendations for clinical practice were graded from A to D (Tables 3, 4).

**Table 3:** Level of evidence

Level I	High-quality prospective randomized clinical trial
Level II	Prospective comparative study
Level III	Retrospective case control study
Level IV	Case series
Level V	Expert opinion

**Table 4:** Grades of recommendation (given to various treatment options based on the level of evidence supporting that treatment)

Grade A	Treatment options are supported by strong evidence (consistent with level I or II studies)
Grade B	Treatment options are supported by fair evidence (consistent with level III or IV studies)
Grade C	Treatment options are supported by either conflicting or poor quality evidence (level IV studies)
Grade D	When insufficient evidence exists to make a recommendation

## Results

**Fig. 1:**
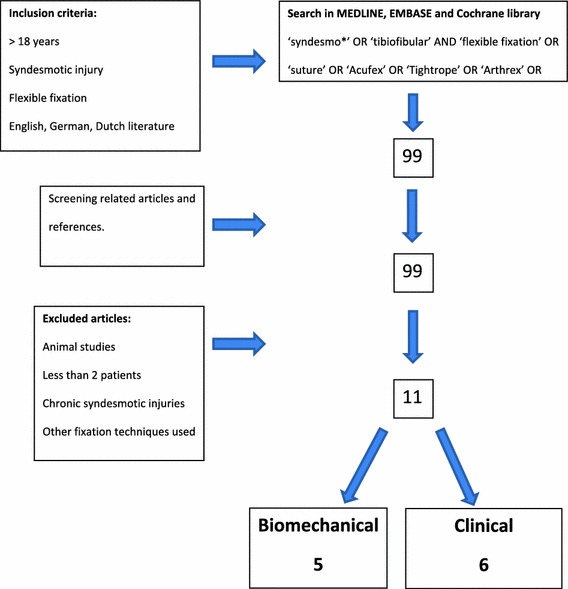
Flowchart summarizing the selection of relevant articles

Ninety-nine publications met the search criteria. After applying the exclusion criteria, 11 articles (five biomechanical and six clinical) were available for data extraction. Pooling of the data was not realistic due to heterogeneity of patient populations, the outcome measures and follow-up (Fig. 1).

### Biomechanical

Five biomechanical studies were included involving 90 cadaveric ankles, ranging from a minimum of eight cadaveric ankles to a maximum of 26 ankles [10, 17, 22, 35, 37]. The first was published in 2005 and the most recent in 2011. The characteristics of these studies are shown in Table 1.

The ankles were tested in different ways: external/internal rotation; dorsal/plantar flexion; eversion/inversion; pull out strength along the axis of the repair apparatus; and axial loading. Although most of the biomechanical studies report there is good resistance to axial and rotational loads, resistance to failure and with restoration of physiologic syndesmotic motion, Forsythe et al. [10] reported inadequate reduction in the syndesmosis at all forces applied. Soin et al. [35] showed similar fibular motions for the suture-button group and the syndesmotic screw group, with neither able to restore native ankle motion.

Forsythe et al. [10] studied external rotation force in two groups (4.5-mm cortical screws versus suture-button). Diastasis at different rotation forces was measured. The screw group retained syndesmotic reduction comparable to the intact specimen at both 2.5 and 5 Nm of loading. The suture-button (fibre wire) group was unable to maintain reduction at the initial load of 2.5 Nm as compared with intact specimens. Both groups were tested to a maximum load of 12.5 Nm moment; beyond this load many of the ankles failed.

Thornes et al. [37] and Klitzman et al. [17] did not have any failures in constructs in either group. Klitzman et al. [17] found that the syndesmotic gap after cycling was not significantly different between the intact group and suture-button group. The screw fixation group had a significantly smaller gap as compared to the two other groups. With increasing external rotation torque, up to 8 kg (20 Nm), Thornes et al. [37] found that the mean diastasis increased gradually in both groups, and these differences were not statistically significant. The suture-button group did give a more consistent performance, a more gradual increase in diastasis compared to the screw fixation.

In the cadaver study by Soin et al. [35], both the suture-button and the screw fixation samples were tested until failure. The screw fixation group had a significantly higher failure torque (median 26.5 Nm, *p* = 0.02) than did the suture-button group (median 23.6 Nm). None of the failures in the suture-button group were due to hardware failure; the screw became bent in three cases of the screw group, but the higher failure torque in the screw group has to be taken into account.

Miller et al. [22] tested the pull out strength 2.0 and 5.0 cm above the tibial plafond. There was no significant difference in strength or displacement between the suture-button and the tricortical screw at either 2.0 or 5.0 cm. The fixation at 5.0 cm had significantly increased holding strength over fixation at 2.0 cm, and the fixation had a significant greater displacement at 2.0 cm than at 5.0 cm.

### Clinical

The clinical studies involved 149 ankles in six studies, with four of these studies being case series [6–8, 36, 38, 42]. The studies demonstrated high functional results with the AOFAS score; there was faster rehabilitation with the suture-button than with screw fixation. Table 2 shows the characteristics and general conclusions of these studies. No major complications were reported in these series, and all authors described the technique as easy and quick to perform. Complications seen in the suture-button group mostly involved hardware removal due to local irritation of the overlying soft tissue [8, 42]. Twelve and sixteen screws had to be removed in the studies by Thornes et al. [38] and Cottom et al. [7], respectively, as compared to none of the suture-buttons. In the study by Thornes et al. [38], no well-defined reasons for screw removal were given, whereas in the study by Cottom et al. [7], the major reasons for removal were screw loosening and screw breakage.

## Discussion

We initiated this work to review the literature on restoration of syndesmotic stability by a suture-button device and to formulate recommendations for clinical practice and future research. The questions were as follows: Is the suture-button fixation secure? Is it efficient? Is it cost-effective?

The distal tibiofibular syndesmosis is not a static structure. When loaded, in gait or passive movement, the syndesmosis allows for three-dimensional motion [1, 3, 26, 31]. Suture-button stabilization of the syndesmosis is thought advantageous as physiologic ankle motions are possibly better preserved, and this potential benefit may lead to better functional outcome. In the article by Thornes et al. [38], patients with suture-button fixation showed better scores in AOFAS scale compared to the screw fixation group and had returned to work earlier. Conversely, Cottom et al. [8] compared both groups and did not find any significant differences in time to postoperative weight-bearing or subjective outcome scores between both groups.

The syndesmotic screw is removed 8–10 weeks after placement usually. This is a second operation with additional cost, new functional limitations and a risk of infection [15, 27, 28, 30, 34]. This contrasts with discussion over the removal of screws; since the publication of a study by Hamid et al. [13], it was shown that a broken screw gives better AOFAS scores than those who had screws removed. Added to this debate, hardware complaints and subsequent need for the removal of the suture-button have been reported [8, 42]. As such, it is not yet clear how this potential benefit should be valued.

This review of the literature suggests some good potential for use of the suture-button in syndesmotic instability. Although the studies are heterogeneous, both clinical (from level II to level IV) and biomechanical (different sources of cadaveric models, different testing), most articles show encouraging results with greater physiologic motion, a good functional outcome and few complications.

### Security and effectiveness

Forsythe et al. [10] were the only investigators who reported a poor outcome from the suture-button in biomechanical testing. They concluded that there was poor ability of the suture to maintain syndesmotic reduction. The authors acknowledged that the ankles may have been tested in supra-physiologic conditions (external rotation torque up to 12.5 Nm). This is confirmed by Shoemaker et al. [33] who described 7.5 Nm to be the external rotation torque that was 75 % of the value reported to elicit discomfort in vivo. Nevertheless, with even lower values of external rotation torque, they found a significant better outcome in the syndesmotic screw group. We agree the authors have tested the ability to retain syndesmotic reduction during external rotation but used forces of a magnitude that was not physiologic. Therefore, the relevance of testing stability of the syndesmotic reduction during external rotation and with forces of such magnitude is questionable.

The other biomechanical studies showed a good ability of the suture-button implant to retain syndesmotic reduction. They revealed also another advantage of this type of fixation which was the preservation of physiologic tibiofibular movement [16, 37]. In contrast, Tornetta et al. [39] showed in their biomechanical study that this does not lead to reduced range of motion, whereas Klitzman et al. [17] described a smaller syndesmotic gap after screw fixation.

From clinical studies, Cottom et al. [7] found no significant difference in clinical outcome at 8–11-month follow-up but a slight but significant difference was found in the tibiofibular clear space from radiological evaluation. The clinical relevance of this observation is not clear, and as stated by Beumer et al. [3], quantitative measurement of syndesmotic parameters in repeated ankle radiographs may be of little value. The limited follow-up in this study prevents any conclusive statements over the long-term results.

In a level III retrospective cohort study by Thornes et al., the AOFAS scores after 3 months and 1 year were significantly higher in the suture-button group than in patients with a conventional screw. They reported a faster return to work. Although the higher AOFAS scores at 3 months may have been influenced by the earlier weight-bearing ability in the ‘flexible’ fixation group, this difference, which remained at the 1 year follow-up, was unlikely to have been influenced by weight-bearing at that late stage [38]. None of the patients treated with suture-button fixation required removal compared with 75 % of patients treated with screw fixation.

All case series (clinical and biomechanical) comment on the speed and ease of the suture-button fixation technique, making it no more complicated than standard screw stabilization. Although no major complications were seen in any of the clinical studies, two authors reported on the need of suture-button removal from local irritation.

Little is known about the long-term outcome of the suture-button fixation since it is a fairly new procedure and the longest available follow-up is only 20 months. Long-term results are needed to confirm the early promising results for the future.

### Cost-effectiveness

One of the advantages attributed to the suture-button technique is the absence of the need for a second operation to remove hardware. Suture-button stabilization seems to be more expensive due to costs of the implant (343 euro for Tightrope Arthrex, 38 euro for conventional Synthes stainless screw, 50 mm). As there is no need for second surgery, which is practised in most hospitals after syndesmotic fixation with a screw, the cost difference will be less. This advantage becomes less important as some authors advocate suture-button removal and others, who practice screw fixation, advocate leaving the screw in situ [8, 13, 42].

The clinical case series report a good functional outcome and early return to work for the suture-button groups, but the design of the included studies prevents conclusive statements to be made based on the available data.

## Conclusion

### Recommendations for clinical practice

The reported advantages of suture-button fixation as compared with traditional stabilization with a screw in repairing a syndesmotic injury include the ability for immediate postoperative weight-bearing, a more physiologic movement at the tibiofibular joint and avoidance of a second operation for hardware removal. The current literature conveys agreement that the technique for placement of a suture-button is easy and quick and the early weight-bearing allowed facilitates a quicker return to work. Whilst the costs are substantially higher, the short-term clinical results are good but there are no long-term results known.
